# Hyperpolarized [1-^13^C]-acetate Renal Metabolic Clearance Rate Mapping

**DOI:** 10.1038/s41598-017-15929-x

**Published:** 2017-11-22

**Authors:** Emmeli F. R. Mikkelsen, Christian Østergaard Mariager, Thomas Nørlinger, Haiyun Qi, Rolf F. Schulte, Steen Jakobsen, Jørgen Frøkiær, Michael Pedersen, Hans Stødkilde-Jørgensen, Christoffer Laustsen

**Affiliations:** 1 MR Research Centre, Aarhus University Hospital, Palle Juul-Jensens Boulevard 99, 8200 Aarhus N, Denmark; 2 Comparative Medicine Lab, Aarhus University Hospital, Palle Juul-Jensens Boulevard 99, 8200 Aarhus N, Denmark; 3 GE healthcare, Freisinger Landstraße 50, 85748 Munich, Germany; 4 Department of Nuclear Medicine and PET Center, Aarhus University Hospital, Nørrebrogade, 8000 Aarhus C, Denmark

## Abstract

^11^C-acetate is a positron emission tomography (PET) tracer of oxidative metabolism, whereas hyperpolarized ^13^C-acetate can be used in magnetic resonance imaging (MRI) for investigating specific metabolic processes. The aims of this study were to examine if the kinetic formalism of ^11^C-acetate PET in the kidneys is comparable to that of ^13^C-acetate MRI, and to compare the dynamic metabolic information of hyperpolarized ^13^C-acetate MRI with that obtained with ^11^C-acetate PET. Rats were examined with dynamic hyperpolarized ^13^C-acetate MRI or ^11^C-acetate PET before and after intravenous injection of furosemide, a loop diuretic known to alter both the hemodynamics and oxygen consumption in the kidney. The metabolic clearance rates (MCR) were estimated and compared between the two modalities experimentally *in vivo* and in simulations. There was a clear dependency on the mean transit time and MCR for both ^13^C-acetate and ^11^C-acetate following furosemide administration, while no dependencies on the apparent renal perfusion were observed. This study demonstrated that hyperpolarized ^13^C-acetate MRI is feasible for measurements of the intrarenal energetic demand via the MCR, and that the quantitative measures are correlated with those measured by ^11^C-acetate PET, even though the temporal window is more than 30 times longer with ^11^C-acetate.

## Introduction

Renal oxygen consumption is closely correlated with tubular sodium reabsorption^1,2^ and is altered by several pathophysiological conditions, including acute kidney disease, ischemic and diabetic nephropathy, and hypertension^3,4^. Dynamic Nuclear Polarization (DNP) magnetic resonance imaging (MRI) is based on the very strong MR-signal from hyperpolarized carbon-13 nuclei inserted in biological molecules. DNP MRI has recently been established as a suitable method for measuring important renal metabolites in various pathophysiological conditions, including diabetes^5–12^, acute kidney injury^13–15^, and acute functional changes^16–18^. Hyperpolarized ^13^C-acetate MRI has been applied to examinations of both rodents and porcine models^19–23^; however, the acquisition and subsequent quantification is challenging at clinical field strengths due to the small chemical shift difference between acetate and the downstream products^24–26^. It is important to note that hyperpolarized ^13^C metabolic flux analysis is currently not fully quantitative and is therefore said to be apparent in nature, thereby limiting the quantitative information available and highlighting the need for new quantitative analysis methods to improve the diagnostic capabilities of the method^27^.

A highly successful clinical metabolic imaging modality, positron emission tomography (PET), similarly relies on the isotopic labeling of biological molecules and allows quantifiable perfusion, uptake, and metabolism^28^. Several metabolically inactive ^13^C-biomarkers, such as ^13^C-urea, have been shown to be particularly useful in perfusion studies, which enable quantitative evaluation^11,14,18,29^ and are analogous to PET perfusion assessment with molecules such as ^15^O-water PET and ^13^N-ammonia PET, although the signal decay is several orders of magnitude faster with hyperpolarized MRI^30,31^. Moreover, in PET, metabolically active molecules rely solely on signal changes associated with the injected compound to quantify various metabolic alterations *in vivo*, as opposed to hyperpolarized ^13^C, where the downstream metabolic conversion is directly detected. One metric, which has been demonstrated to be associated with alterations in oxidative metabolism, is the metabolic clearance rate (MCR) of ^11^C-acetate PET^30,32,33^. ^11^C-acetate is quickly extracted from plasma by the renal tissue and accumulates approximately in proportion to the renal blood flow (RBF)^29^:

1 $$RBF=\frac{RBV}{MTT}$$

where RBV is the renal blood volume and MTT is the mean transit time in the renal parenchyma. Once inside the cell, ^11^C-acetate will be metabolized via acetyl-CoA synthetase to acetyl-CoA, which enters the tri-carboxylic acid (TCA) cycle, a reaction that has been shown to be proportional to the oxidative rate of the cycle^30^.

Juillard *et al*.^34^ demonstrated that the renal ^11^C-acetate MCR, mono-exponential clearance, was significantly correlated with renal oxygen consumption using ^11^C-acetate PET. It was concluded that renal ^11^C-acetate MCR would be of value in monitoring the effects of interventions and in understanding the pathophysiology of chronic renal diseases. MCR, denoted *K* (in units min^−1^) in ^11^C-acetate PET, estimated from the decay/removal (signal leaving the compartment in question and/or metabolic conversion), is considered to be a quantitative measure of oxidative metabolism^30,32,33^. The clearance rate is then determined by fitting the tissue dynamic curve with a one-compartment model, single-exponential fit. Alternatively, a novel method, which utilizes the relationship between the mean transit time and the MCR, has been proposed to allow similar quantitative clearance rate mapping^30,35^:

2 $$K=\frac{1}{MTT}$$

Denoted hereafter as *K*_*MTT*_. Analogously to ^11^C-acetate, we investigated [1-^13^C]-acetate hyperpolarized MRI as a novel method for measuring renal oxygen metabolism solely using the [1-^13^C]-acetate signal, rather than separating the metabolic by-products as typically performed with hyperpolarized MRI, by utilizing this simple MTT relationship.

The aims of this study were to investigate the feasibility of hyperpolarized ^13^C-acetate MRI for measuring the MCR in the kidneys before and after administration of a diuretic, and to compare the findings with ^11^C-acetate MCR PET findings^35^, *in vivo* and in simulations.

## Methods and Materials

### Simulations

**Figure 1:**
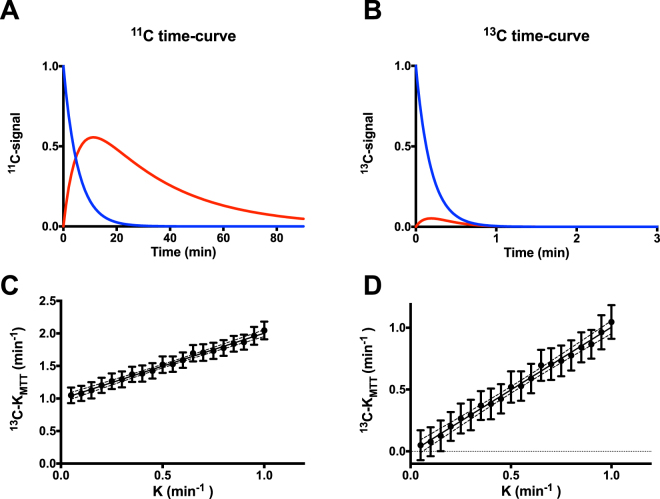
^11^C-acetate and hyperpolarized ^13^C-acetate PET simulations. (**A**) ^11^C-acetate kinetic transit from one compartment (blue curve) to another compartment (red curve), taking the radioactive tracer decay of 20.4 min into account. (**B**) Similar metabolic clearance rate (K = 0.15) of the hyperpolarized ^13^C-acetate kinetic transit from one compartment to another (red curve × 5), taking into account T_1_ relaxation (14 s) and RF depletion (10 degrees). Note 3 min versus 90 min in the PET examination. (**C**) *In silico* estimation of the estimated *K*_*MTT*_ from the tissue curve (blue curve, Fig. 1B), taking decay into account, RF inhomogeneity (7.5–12.5°), T_1_ variation of 12–16 s, and random noise of 5% variation resembling acquisition noise, showing a linear correlation (*P* < 0.0001, R^2^ = 0.2) between the actual metabolic clearance rate *K* and the estimated mean transit time metabolic clearance rate *K*_*MTT*_, using T_1_ = 14 s as the correction factor (linear regression: K_MTT_ = 1.0 K + 0.99). (**D**) Offset corrected (K_MTT_-0.99) found by linear regression, showing good agreement between the values recorded by hyperpolarized ^13^C and the *K* input in the simulation. Represented as mean ± confidence intervals. PET, positron emission tomography; RF, radiofrequency field.

In order to investigate the accuracy of the proposed formalisms in determining *K*, we simulated ^11^C and ^13^C acetate metabolic conversion. We assumed that ^11^C-acetate accurately describes the oxidative renal metabolism, and that ^11^C and ^13^C labelled acetate share common pharmacokinetic information, although the radioactivity decay and longitudinal relaxation are significantly different. A two compartment, unidirectional kinetic simulation of ^11^C-acetate and ^13^C-acetate kinetic conversion was performed using MATLAB (MATLAB 2016a, The MathWorks Inc., Natick, MA, USA), with the approximately achieved values *in vivo*: *K* = 0.15 min^−1^, ^11^C-acetate decay = 20.4 min, ^13^C-acetate relaxation time = 14 s at 3 Tesla^24^, and flip angle = 10 degrees, showing a markedly increased conversion over 90 min compared with 3 min (Fig. 1A,B). In order to simulate the *in vivo* situation, a radiofrequency field (RF) inhomogeneity of 50% and T_1_ variation of 29% in concern with a noise level of 5% was used to identify the relationship between the actual MCR (ranging from 0.05–1.0) and the proposed formalism derived K_MTT_ (Fig. 1C,D). Each MCR point represents 90 iterations with 5% noise, covering the RF and T_1_ variation ranges.

### Animals

Eleven healthy rats were included in this study. All animals were anaesthetized with 3% sevoflurane in 2 L/min air as breathing gas. Blood glucose levels were measured from tail capillary blood with a Contour blood glucose meter (Bayer Diabetes Care, Copenhagen, Denmark). Tail vein catheterization (G 24) was performed for administration of hyperpolarized [1-^13^C]-acetate and a diuretic (furosemide). All catheters were flushed with heparinized saline water. Temperature was maintained at 37 **°**C (SA Instruments, Stony Brook, NY, USA). The experiments complied with the Guidelines for the Use and Care of Laboratory Animals, and were approved by the Danish Inspectorate of Animal Experiments (J.nr. 2014-15-0201-00327).

### Hyperpolarized ^13^C-Acetate MRI

Six healthy female Wistar rats (258 ± 8 g) were scanned in a 3 Tesla GE HDx MRI system equipped with a hydrogen/carbon-RF quadrature transmit/receive-coil (GE Healthcare, Milwaukee, WI, USA). [1-^13^C]-acetate was polarized in a SpinLab system (GE Healthcare). The kidneys were localized by a standard gradient-echo sequence, and a slice covering both kidneys was shimmed automatically. An axial oblique slice-selective (10 mm) ^13^C-dynamic single shot spiral (field of view = 80 × 80, matrix = 32 × 32) sequence (120 sec, one image/sec) was initiated at the start of injection. A volume of 1.5 mL [1-^13^C]-acetate was injected into the tail vein over a period of 15 sec. Twenty minutes after a furosemide injection (10 mg/kg), a second dynamic ^13^C-MRI was repeated following an [1-^13^C]-acetate injection (with similar concentration and injection rate). In order to verify the used *in vivo* effective T_1_ time for correction of the *in vivo* data. Whole blood was extracted from two healthy rats into sodium heparin vacuum tubes. The blood was stored at 5 °C. Prior to the experiment the blood was heated and maintained at 37 °C. A volume of 4.5 mL blood was mixed with hyperpolarized ^13^C-acetate (0.5 ml) prior to placement in the scanner. The MR experiment was acquired over 120 s (120 acquisitions), with a constant flip angle of 10°. The single exponential decay was fitted in MATLAB and corrected for RF depletion.

### ^11^C-acetate PET

Five healthy Sprague-Dawley rats (three female, two male; weight: 314 ± 64 g) were scanned twice (90 min per scan) following injection with ^11^C-acetate in a Mediso nanoScan PET/MRI (Mediso, Budapest, Hungary) baseline and again 20 min post furosemide administration. Data were acquired in PET list-mode and reconstructed as 26 frames (90 min: 8 × 15 sec, 8 × 60 sec, 4 × 5 min, 6 × 10 min) with a three-dimensional (3D) iterative algorithm (Tera-Tomo 3D, Budapest, Hungary), full detector model, and normal regularization (Mediso, Budapest, Hungary) involving four iterations and six subsets, and a voxel size of 0.4 × 0.4 × 0.4 mm^3^ (0.064 mm^3^). Data were corrected for random coincidence events using a delayed coincidence window, and further corrected for dead time and decay. Images were corrected for attenuation and scatter using 18-min long 3D MR gradient echo sequences (TR 2.0 ms, TE 2.1 ms, flip angle 25°, 0.5 mm slice thickness, and horizontal orientation). In summary, the renal ^13^C-acetate and ^11^C-acetate distribution was measured under baseline physiological conditions, and again after a furosemide induced reduction of the active oxygen-dependent sodium transport in the ascending loop of Henle.

### Analysis

MRI/PET data were imported to the Osirix software (Pixmeo, Geneva, Switzerland), and regions-of-interests on the left and right kidney parenchyma were manually segmented in order to measure the mean renal activity-curve. In the hyperpolarized ^13^C-acetate study, an additional region-of-interest was drawn inside the abdominal aorta to obtain the arterial input function.

### PET MCR

Two methods were used to estimate the MCR using ^11^C-acetate: (1) a single exponential fit of the first 10–12 min after the inflow to the cortical tissue, denoted as *K*_*mono*_^34^, and (2) an analogous estimated *K*_*MTT*_, using the inverse of the mean transit time (MTT) in the tissue, estimated from the first-order moment:

3 $${K}_{MTT}=\frac{{\int }_{0}^{\infty }{C}_{ROI}(t)dt}{{\int }_{0}^{\infty }t{C}_{ROI}(t)dt}$$

With *t* being the acquisition intervals and ^11^C-acetate concentration (*C*_*ROI*_) in the region-of-interest.

### Hyperpolarized MCR

In order to account for the first pass perfusion of the 2 min acquisition (90 min in PET), a model-free deconvolution was used (UMMperfusion plugin^36^) to estimate the [1-^13^C]-acetate renal plasma perfusion and metabolic conversion rate (*K*_*MTT*_) in units of min^−1^, using the approximation that MTT is reciprocal similar to *K* (denoted *K*_*MTT*_)^35^ (equation 1). A hematocrit (Hct) of 0.45 was assumed and a 0.15 regularization kernel was used for all analyses. The plasma flow was converted to RBF by dividing the renal plasma flow with (1-*Hct*). In order to account for the depolarization of the hyperpolarized signal (T_1_ and RF depletion), a parametric relationship has previously been demonstrated^29^:

4 $$MTT=\frac{MTT\text{'}}{\beta }$$

where depolarization factor β is defined as:

5 $$\beta =1-\frac{MTT\text{'}}{{T}_{1}}$$

With MTT’ being the measured (uncorrected) MTT and T_1_ being the relaxation time of the hyperpolarized ^13^C-acetate signal (here assumed to be 14 s^24^).

### Statistics

Normality was assessed with quantile-quantile plots. A *P*-value below 0.05 was considered statistically significant. Statistical analysis was performed using GraphPad Prism (GraphPad Software, La Jolla, CA, USA). A paired student’s t-test with equal standard deviation was used for statistical analysis of the pre/post experiments and an unpaired student’s *t*-test with equal standard deviation was used to test the difference between the perfusion measurements.

## Results

**Figure 2:**
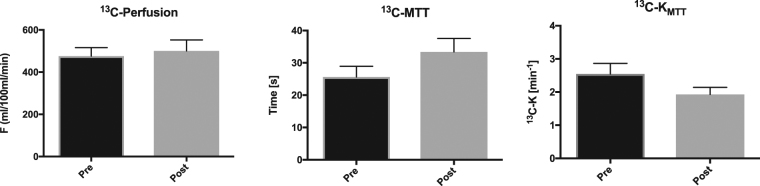
Examples of [1-^13^C]-acetate uptake in the aorta and kidneys over time. Hyperpolarized [1-^13^C]-acetate signal overlaid ^1^H-anatomical MR images of an axial slice, showing two kidneys and the presence of a signal in the aorta and following the kidneys. MR; magnetic resonance.

**Figure 3:**
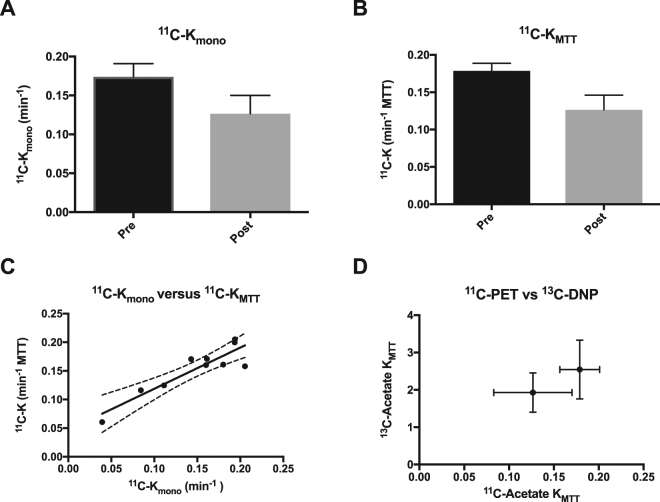
^13^C-acetate *in vivo* hemodynamic parameters. Acetate perfusion (min/100 ml/mL), mean transit time (MTT) (sec), and acetate mean transit time metabolic clearance rate *K*_*MTT*_ (min^−1^) before and after administration of furosemide. The mean is plotted with standard errors.

The simulated single ^11^C-acetate compartment response was found to correlate well with the inverse MTT derived MCR ^11^C-*K*_*MTT*_ both with (*P* < 0.0001, R = 0.99) and without (*P* < 0.0001, R = 0.99) decay correction (*data not shown*). The ^13^C-acetate compartment response was found to correlate similarly well without both relaxation and RF depletion (*P* < 0.0001, R = 0.99), although an offset was observed between the *K* input in the simulation and the estimated ^13^C-K_MTT_. A similar offset correlation was recorded taking into account relaxation, RF depletion, and inhomogeneity, as well as experimental variation (*P* < 0.0001, R^2^ = 0.2; Fig. 1). The linear relationship between the simulated input *K* value and the estimated noise simulations identified an offset of approximately 1 in the estimated ^13^C-K_MTT_ compared with the *K* input in the simulation. All rats were scanned in the fed state, and blood glucose levels were 7.3 ± 0.8 (±SD) mmol/L. Hyperpolarized [1-^13^C]-acetate showed accumulation in both kidneys following arterial filling (Fig. 2). The ^13^C-acetate RBF did not differ statistically between pre (476 ± 97 ml/100 ml/min) and post administration of furosemide (501 ± 127 (±SD) ml/100 ml/min; paired *t*-test: *P* = 0.63; Fig. 3). Furthermore, we found a significantly different ^13^C-acetate MTT of 26 ± 8.1 (±SD) sec at baseline compared with 33.4 ± 10.1 (±SD) sec post furosemide administration (paired *t*-test: *P* = 0.05). Similarly, there was a significant difference in the ^13^C-*K*_*MTT*_ between baseline 2.5 ± 0.8 (±SD) min^−1^ and post furosemide administration 1.9 ± 0.5 (±SD) min^−1^ (paired *t*-test: *P* = 0.03) (Fig. 3). In order to confirm the *in vivo* effective T_1_, an *ex vivo* experiment on whole blood was performed. A whole blood ^13^C-acetate T_1_ of 18 ± 0.2 (±SD) was found.

**Figure 4:**
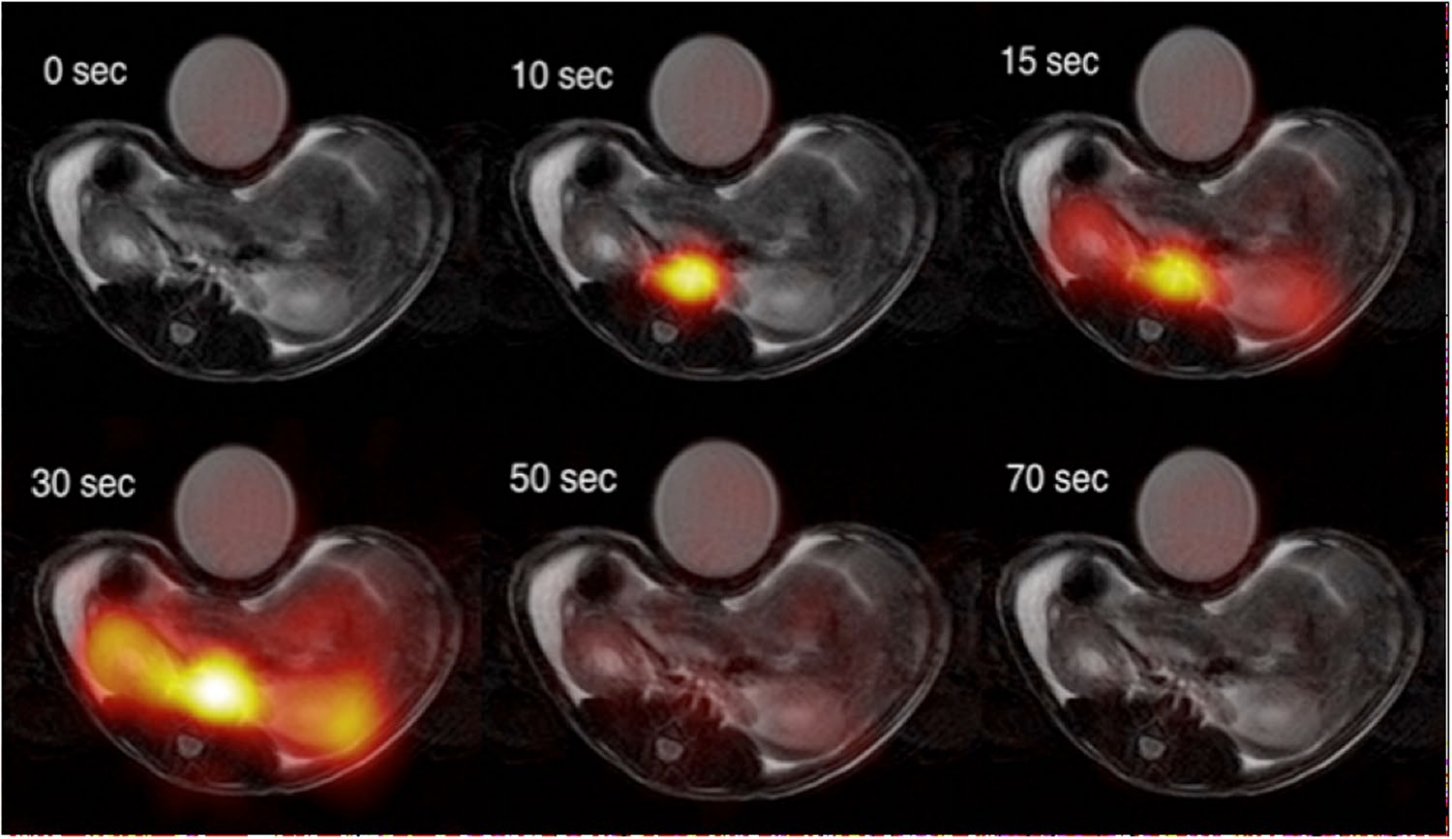
Examples of ^11^C-acetate uptake in the aorta and kidneys over time. Positron emission tomography ^11^C-acetate signal overlaid ^1^H-anatomical MR images of a coronal slice, showing two kidneys and the presence of a signal in the aorta and following the kidneys. MR; magnetic resonance.

**Figure 5:**
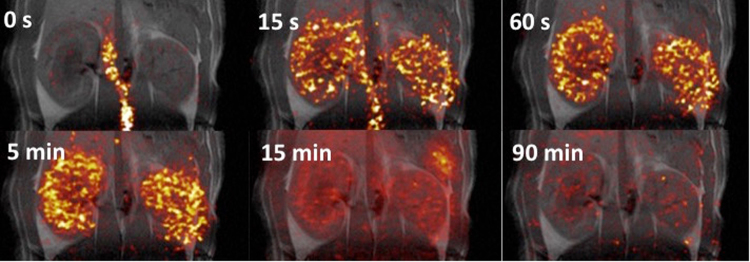
^11^C-acetate i*n vivo* kinetic parameters. (**A**) ^11^C-acetate single exponential metabolic clearance rate, K_mono_. (**B**) ^11^C-acetate mean transit time metabolic clearance rate, *K*_*MTT*_. (**C**) Correlations between the decay derived or the first moment derived rates and the hyperpolarized ^13^C, showing a positive correlation (R^2^ = 0.82, *P* = 0.0003). (**D**) A tendency towards a similar response to furosemide treatment is seen between the ^11^C-PET and the ^13^C-hyperpolarization estimations. The mean is plotted with standard errors. PET, positron emission tomography.

In order to verify the relationship between ^11^C-K_mono_ and ^11^C-K_MTT_, *in vivo* ^11^C-acetate dynamic PET images were acquired over 90 min prior to and post furosemide administration (Fig. 4). We observed a statistically significant decrease of 29% (paired *t*-test: *P* = 0.01) for ^11^C-*K*_*MTT*_ estimated from the MTT, and a similar numerical decrease of 27% for ^11^C-*K*_*mono*_, although the difference was not significant (paired *t*-test: *P* = 0.21) *in vivo* (Fig. 5). Furthermore, a positive correlation was observed between ^11^C-PET K_mono_ and ^11^C-K_MTT_ (*P* = 0.01, R^2^ = 0.84) (Fig. 5). No significant difference was observed in the fractional reduction of ^13^C-K_MTT_ (30 ± 16%) and ^11^C- K_MTT_ (22 ± 16%) (paired *t*-test: *P* = 0.46) (Fig. 5).

## Discussion

This study investigated the feasibility of hyperpolarized ^13^C-acetate MRI for measuring the MCR in kidneys before and after administration of a diuretic, and compared the findings with those of ^11^C-acetate MCR PET *in vivo* and in simulations. The main finding of this study was that it was possible to measure the renal MCR changes associated with furosemide treatment using hyperpolarized ^13^C-acetate, and that these changes correlated with the values reported for quantitative ^11^C-acetate PET^34^. The method is based only on the injected ^13^C-acetate signal itself, without the need to sample the downstream metabolic products.

Our findings support the use of ^11^C-acetate mono-exponential decay analysis in the investigation of renal oxidative alterations associated with changes in sodium reabsorption induced by furosemide, and show that the MCR can be accurately estimated using the ^11^C-acetate transit time in the renal parenchyma. Similarly, the hyperpolarized ^13^C-acetate is able to show a similar relationship, although there is an offset due to the short temporal window, and fast decaying signal due to RF depletion and signal relaxation decay.

The renal perfusion estimated with ^13^C-acetate hyperpolarization was similar to previously reported values with dynamic contrast enhanced imaging in rats^37^ and with hyperpolarized ^13^C-2-hydroxyethylacrylate^38^. Comparing ^13^C-acetate perfusion and MTT to the metabolically inactive hyperpolarized ^13^C-urea under similar conditions (see Supplemental Fig. 1) results in similar perfusion characteristics pre and post furosemide administration, while no change is seen in the ^13^C-MTT between baseline and furosemide challenge for ^13^C-urea^11^, supporting metabolic conversion of ^13^C-acetate to be the origin of the changes seen in this study. Previous studies have demonstrated time-dependent renal perfusion alterations following administration of furosemide^39–41^. In the present study, no alterations in renal perfusion post furosemide were observed. This could be due to the timing or the low spatial resolution, making spatial localization difficult. An increased RBF post furosemide administration would be expected to limit the renal oxygen consumption measurements and therefore potentially mask the altered oxygen consumption.

The model-free deconvolution has previously been applied in hyperpolarized experiments, yielding accurate ^13^C-MTT maps with a minor deviation originating from the relaxation decay of the tracer^29^. These results are further supported by the recent hyperpolarized water perfusion in the porcine kidney^42^, demonstrating that the accurate use of formalism can determine the perfusion of the kidney.

The increased conversion rate of [1-^13^C]-acetate observed following furosemide administration supports the view that this diuretic drug increased the filling of the cortical space, while the overall energetic demand was reduced due to the reduced sodium reabsorption induced by furosemide treatment. This finding is consistent with a previous experiment performed with hyperpolarized ^13^C-urea in the porcine kidney^16^. The hyperpolarized signal had a significantly shorter decay compared with ^11^C-acetate.

The *in vivo* ^13^C-acetate MCR (prior to offset correction) in this study was two orders of magnitude larger than that reported with radioactive ^11^C-acetate in the porcine kidney^34^ and the *in vivo* ^11^C-acetate MCR values reported in this study. This discrepancy may be explained by the large difference in signal decay, which was more than twenty times longer for ^11^C-acetate compared with ^13^C-acetate, and the RF depletion following repetitive excitations, showing a similar offset to the estimated ^13^C-K_MTT_. This was supported by the simulations, showing a remarkable consistency with the *in vivo* results. Additionally, arterial input function and renal perfusion are expected to affect the hyperpolarized signal to a large degree due to the short time frame of the investigation.

Bi-exponential ^11^C-acetate PET MCR has previously been described in the myocardium, with the fast decaying component correlating with myocardial oxygen consumption^30^. This is in contrast to the findings of the present study and the study by Juillard *et al*.^34^, showing a single exponential dependency and an approximately 10 times lower MCR. One contributing factor to this difference could partly originate from the difference in renal and myocardial metabolism, which is supported by the two-fold increase in the acetate-to-acetylcarnitine in rat heart compared with the kidney^24^. While MCR intra-species differences were expected, similar ^11^C-acetate MCR have been reported among porcine, rabbit, and rat studies^34,43^. The reason for this intra-species similarity in MCR is currently unknown.

A potential limitation of the ^13^C-acetate interpretation was the relaxation decay, which is fast and difficult to measure *in vivo* because the different cellular compartments exhibit variations in relaxation properties^44^. Furthermore, intra-renal ^13^C relaxation differences have been demonstrated in the rodent kidney, both intra-voxel and across the kidney, indicating further improvements in the quantification by adequately accounting for these intra-renal relaxation differences^9,17,45,46^.

In the present study, we utilized a simple parametric relationship to correct for the T_1_ relaxation (here a simple single component), allowing already existing software packages to be utilized without any modifications^36,37^. The ^13^C-acetate T_1_ relaxation time (estimated renal T_1_ relaxation time^24^) used for the correction was similar to previously reported values at 3 T *in vivo* ^21,24,47^ and largely similar to the whole blood ^13^C-acetate T_1_ at 3 T (using the whole blood T_1_ for correction will results in a slightly underestimated perfusion assessment, see Supplemental Fig. 2). A similar fast *in vivo* relaxation have been demonstrated at 9.4 T^48^, although it is important to note that reported values in solution is typically more than 40 s and even longer at lower fields^47,49–51^. This is likely due to tissue specific effects on the *in vivo* T_1_, compared to *ex vivo* blood. It’s unlikely that the T_1_ relaxation of the ^13^C-acetate metabolic breakdown products affects the MCR found here, as ^13^C-acetate metabolic observable signal at 3 T is very small^24^. This can easily be solved by separating the different metabolic components by either selective excitation or spectroscopic separation and thus improving both the MCR and T_1_ relaxation estimations^52,53^.

An offset in the estimated MCR originating from the shorter imaging window (offsetting the accurate determination of *K*_*MTT*_) with hyperpolarized ^13^C compared with ^11^C PET, was observed in the ^13^C-acetate simulations and *in vivo* ^13^C-acetate experiments. Using a simple offset correction, the ^13^C-K_MTT_ results in a similar MCR value, thus supporting the inter-relationship of ^11^C and ^13^C, although the time span was significantly different. This supports the use of ^11^C-PET for improving the quantification of ^13^C hyperpolarized MRI examinations. Using a similar offset found in the simulations, an overestimation of the *in vivo* results was identified using hyperpolarized ^13^C. This could be due to the difference in input function, T_1_ relaxation, animal gender, and strain, in combination with the fact that the estimation was performed independently, indicating that simultaneous hyperpolarized MR and PET examinations are needed to describe the inter-relationship of ^11^C and ^13^C.

## Conclusion

This study introduced a novel hyperpolarized ^13^C-acetate MRI method for the investigation of renal MCR, which is a potential surrogate marker of oxidative metabolism. Furthermore, we found that the prior developed formalism for renal imaging with PET tracers allowed for simple and intuitive processing of ^13^C single metabolite concentration curves. The formalism is not limited to ^13^C-acetate imaging as such and can easily be extended to cover the more typical hyperpolarized biomarkers such as [1-^13^C]-pyruvate. This study suggests a link between the metabolic information obtained with PET and hyperpolarized MRI. However, further investigations are needed to fully determine the relationship between the MCR determined with ^11^C-PET and ^13^C-hyperpolarized MRI.

## Electronic supplementary material

Supplemental information
