# Publisher Correction: Hyperpolarized [1-^13^C]-acetate Renal Metabolic Clearance Rate Mapping

**DOI:** 10.1038/s41598-018-29057-7

**Published:** 2018-07-20

**Authors:** Emmeli F. R. Mikkelsen, Christian Østergaard Mariager, Thomas Nørlinger, Haiyun Qi, Rolf F. Schulte, Steen Jakobsen, Jørgen Frøkiær, Michael Pedersen, Hans Stødkilde-Jørgensen, Christoffer Laustsen

**Affiliations:** 1 MR Research Centre, Aarhus University Hospital, Palle Juul-Jensens Boulevard 99, 8200 Aarhus N, Denmark; 2 Comparative Medicine Lab, Aarhus University Hospital, Palle Juul-Jensens Boulevard 99, 8200 Aarhus N, Denmark; 3 GE healthcare, Freisinger Landstraße 50, 85748 Munich, Germany; 4 Department of Nuclear Medicine and PET Center, Aarhus University Hospital, Nørrebrogade, 8000 Aarhus C, Denmark

Correction to: *Scientific Reports* 10.1038/s41598-017-15929-x, published online 22 November 2017

This Article contains an error in the order of the Figures. Figures 2, 3, 4 and 5 were published as Figures 4, 2, 5 and 3 respectively. The correct Figures 2, 3, 4 and 5 appear below. The Figure legends are correct.

**Figure 2:**
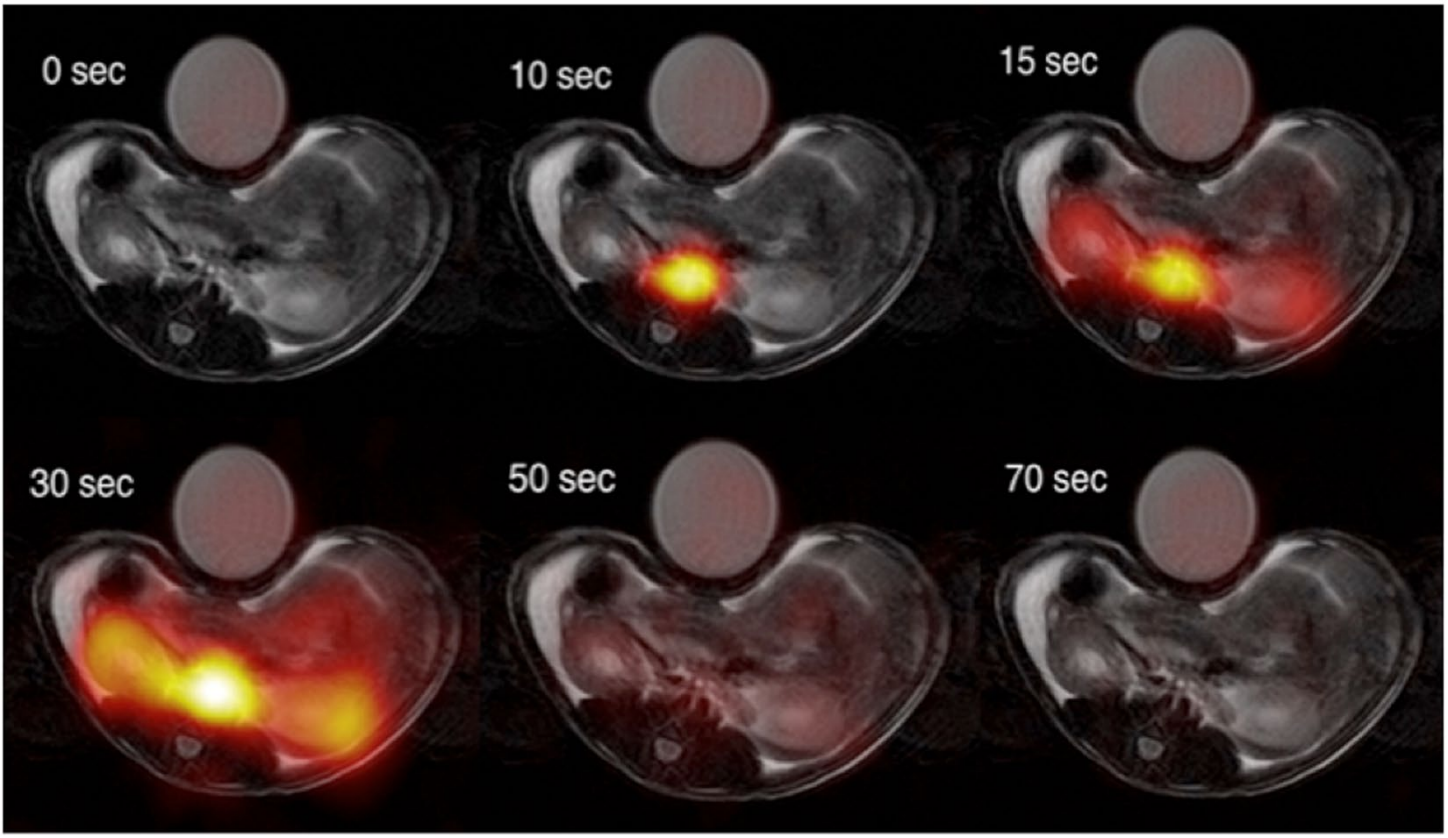
Examples of [1-^13^C]-acetate uptake in the aorta and kidneys over time. Hyperpolarized [1-^13^C]-acetate signal overlaid ^1^H-anatomical MR images of an axial slice, showing two kidneys and the presence of a signal in the aorta and following the kidneys. MR; magnetic resonance.

**Figure 3:**
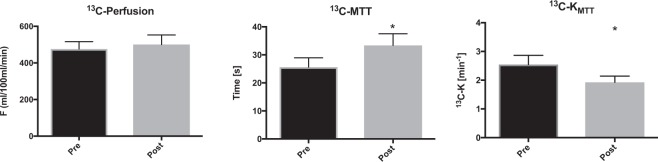
^13^C-acetate *in vivo* hemodynamic parameters. Acetate perfusion (min/100 ml/mL), mean transit time (MTT) (sec), and acetate mean transit time metabolic clearance rate *K*_*MTT*_ (min^−1^) before and after administration of furosemide. The mean is plotted with standard errors.

**Figure 4:**
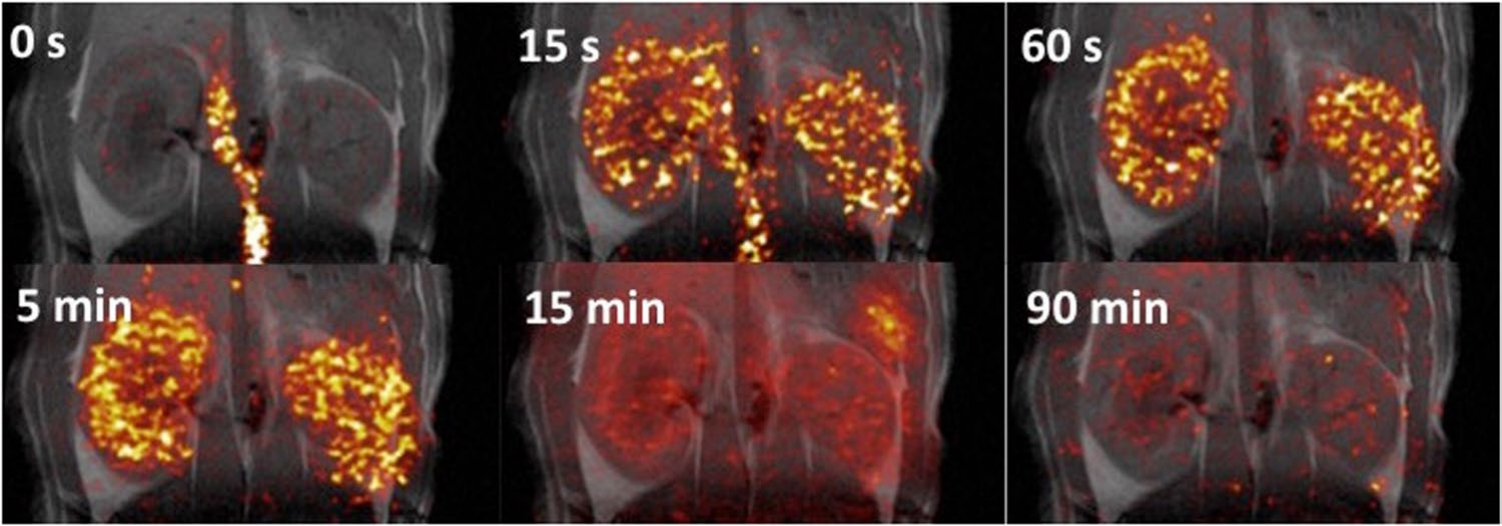
Examples of ^11^C-acetate uptake in the aorta and kidneys over time. Positron emission tomography ^11^C-acetate signal overlaid 1H-anatomical MR images of a coronal slice, showing two kidneys and the presence of a signal in the aorta and following the kidneys. MR; magnetic resonance.

**Figure 5:**
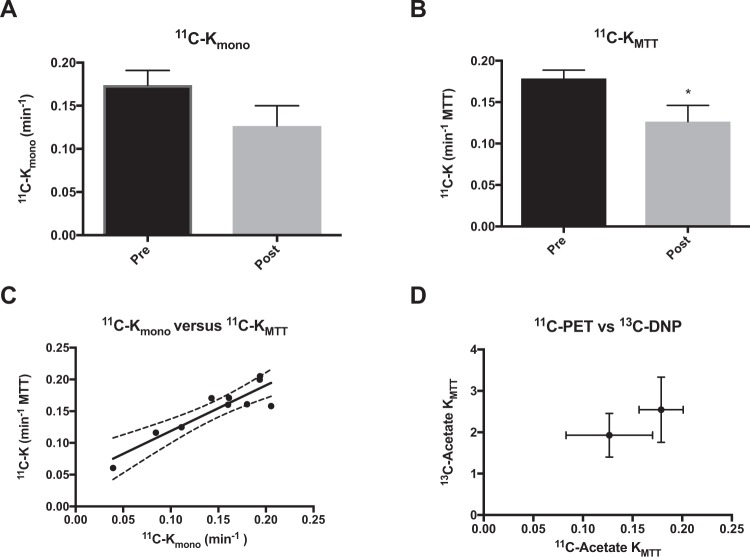
^11^C-acetate *in vivo* kinetic parameters. (**A**) ^11^C-acetate single exponential metabolic clearance rate, K_mono_. (**B**) ^11^C-acetate mean transit time metabolic clearance rate, *K*_*MTT*_. (**C**) Correlations between the decay derived or the first moment derived rates and the hyperpolarized ^13^C, showing a positive correlation (R^2^ = 0.82, *P* = 0.0003). (**D**) A tendency towards a similar response to furosemide treatment is seen between the ^11^C-PET and the ^13^C-hyperpolarization estimations. The mean is plotted with standard errors. PET, positron emission tomography.

