# Supplementary material for: Hyperpolarized [1-13C]-acetate Renal Metabolic Clearance Rate Mapping
Source: Sci Rep. 2017 Nov 22;7:16002. doi: 10.1038/s41598-017-15929-x (PMC5700138; doi:10.1038/s41598-017-15929-x)
Supplement: Supplementary file 1 — Supplemental information [file 41598_2017_15929_MOESM1_ESM.pdf]

Hyperpolarized [1-^{13}C]-acetate Renal Metabolic Clearance Rate Mapping

Emmeli F. R. Mikkelsen[1,2,§], Christian Østergaard Mariager[1,§], Thomas Nørlinger[1,2], Haiyun Qi[1], Rolf F. Schulte[3], Steen Jakobsen[4], Jørgen Frøkiær[4], Michael Pedersen[2], Hans Stødkilde-Jørgensen[1], and Christoffer Laustsen[1]

[1]*MR Research Centre, Aarhus University Hospital, Palle Juul-Jensens Boulevard 99, 8200 Aarhus N, Denmark.*
[2]*Comparative Medicine Lab, Aarhus University Hospital, Palle Juul-Jensens Boulevard 99, 8200 Aarhus N, Denmark.*
[3]*GE healthcare, Freisinger Landstraße 50, 85748 Munich, Germany.*
[4]*Department of Nuclear Medicine and PET Center, Aarhus University Hospital, Nørrebrogade, 8000 Aarhus C, Denmark*

[§]E.F.R.M and C.Ø.M contributed equally to this work

Corresponding author:
Christoffer Laustsen
Palle Juul Jensens boulevard 99
8200 Aarhus N
Denmark
M +45 24439141
Tel: +45 784 56139
Fax: +45 784 56110
Email: cl@clin.au.dk

Financial support: The study was supported by Sundhed og Sygdom, Det Frie Forskningsråd and Aarhus University research fund.

Short running title: Hyperpolarized ^{13}C-acetate MRI

SUPPLEMENTAL FIGURES.

Supplemental figure 1.

In order to verify the existence of a metabolic component in the mean transit time, 4 female rats receiving a similar furosemide treatment was processed with the proposed method. A global T_1 relaxation time of 24 sec, was used to correct the ^{13}C-urea perfusion data.

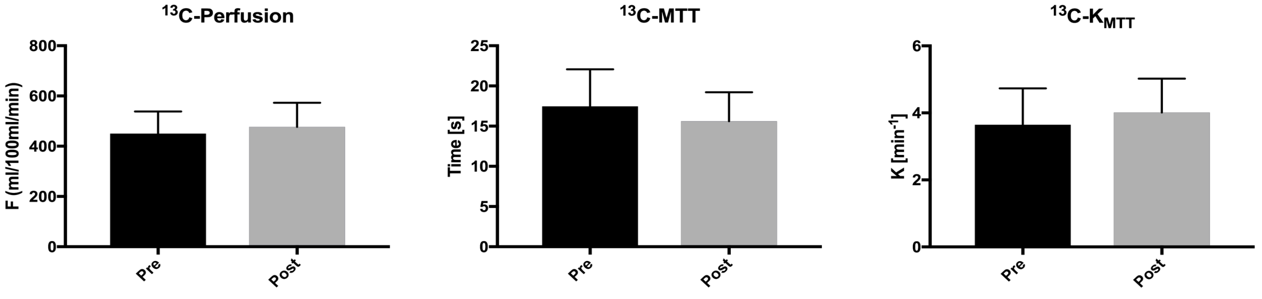

SUPPLEMENTAL FIGURE 1. The ^{13}C-urea RBF did not differ statistically between pre (450 ± 88 ml/100 ml/min) and post administration of furosemide (477 ± 96 (±SD) ml/100 ml/min; paired *t*-test: *P* = 0.7). Furthermore, no difference in the ^{13}C-urea MTT of 18 ± 5 (±SD) sec at baseline compared with 16 ± 4 (±SD) sec post furosemide administration (paired *t*-test: *P* = 0.6) was found.

Supplemental figure 2.

Whole blood was extracted from two healthy rats into sodium heparin vacuum tubes. The blood was stored at 5° C. Prior to the experiment the blood was heated and maintained at 37° C. A volume of 4.5 ml blood was mixed with hyperpolarized ^{13}C-acetate (0.5 ml) prior to placement in the scanner. The MR experiment was acquired over 120 s (120 acquisitions), with a constant flip angle of 10°. The single exponential decay was fitted in MATLAB and corrected for RF depletion.

$$\frac{1}{T_1} = \frac{1}{T_{1eff}} - \frac{\ln\cos\theta}{TR} \quad \text{(supplement eq. 1)}$$

Where θ is the RF flip angle and TR is the repetition time. Using the *ex vivo* T_{1eff} for correction, results in an underestimated absolute perfusion, lower MTT and thus larger K_{MTT}.

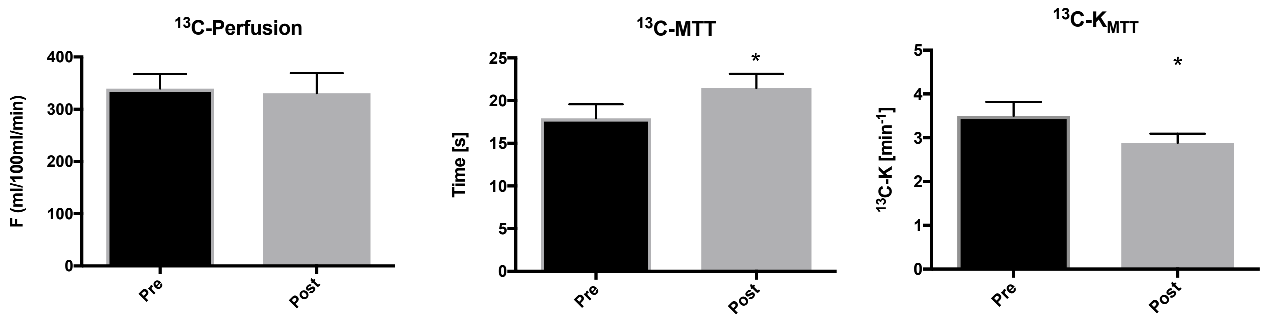

SUPPLEMENTAL FIGURE 2. ^{13}C-acetate *in vivo* hemodynamic parameters (using whole blood T_1 correction).Acetate perfusion (min/100 ml/mL), mean transit time (MTT) (sec), and acetate mean transit time metabolic clearance rate K_{MTT} (min^{-1}) before and after administration of furosemide. The mean is plotted with standard errors.
